# Pre- and Post-treatment Levels of Plasma Metabolites in Patients With Bipolar Depression

**DOI:** 10.3389/fpsyt.2021.747595

**Published:** 2021-12-17

**Authors:** Xiang-Jie Guo, Peng Wu, Xiao-Hong Cui, Jiao Jia, Shuang Bao, Fei Yu, Li-Na Ma, Xiang-Xin Cao, Yan Ren

**Affiliations:** ^1^Department of Forensic Medicine, Shanxi Medical University, Taiyuan, China; ^2^Department of Psychiatry, Shanxi Bethune Hospital, Shanxi Academy of Medical Sciences, Tongji Shanxi Hospital, Third Hospital of Shanxi Medical University, Taiyuan, China; ^3^Tongji Hospital, Tongji Medical College, Huazhong University of Science and Technology, Wuhan, China

**Keywords:** bipolar disorder (BD), bipolar depression, pre- and post-treatment, metabolomics, efficacy evaluation, biomarker

## Abstract

**Background:** Bipolar disorder (BD) is a serious mental disease with complex clinical manifestations and high recurrence rate. The purpose of this study was to detect metabolites related to the diagnosis and efficacy evaluation of bipolar depression in plasma samples by metabolomics.

**Methods:** Thirty-one bipolar depression patients were recruited and completed 8 weeks medication and a matched group of 47 healthy controls (HCs) was recruited. Nuclear magnetic resonance spectroscopy was used to profile plasma samples of bipolar depression patients at baseline and after 8 weeks medication, and HCs. Then Multivariate statistical analysis was performed to analyze differences of plasma metabolites among the three groups.

**Results:** We detected seven specific differential metabolites in bipolar depression. Six of the metabolites were returned to the normal levels in different degrees after 8 weeks medication, only Glycine continuously decreased in the acute and significant improvement stages of bipolar depression (VIP > 1 and *p* < 0.05). These differential metabolites involved several metabolic pathways.

**Limitations:** The small sample size was one of the most prominent limitations. Each BD patient was given an individualized medication regimen according to the clinical guidelines.

**Conclusion:** There were metabolites changes before and after 8 weeks medication. Glycine may be a characteristic marker of bipolar depression and does not change with the improvement of bipolar depression, while other 6 differential metabolites may be biomarkers associated with the pathological development or the improvement of bipolar depression. And, these differential metabolites mainly related to energy metabolism, amino acid metabolism and gut microbiota metabolism.

## Introduction

BD is a chronic severe mental illness, characterized by the intermittent occurrence of depression and mania (or hypomania). The average age of onset of BD was 20–30 years (1), with a lifetime prevalence of about 2.4% (2) and a recurrence rate of 40–50% within 2 years (3). Patients with BD often suffer from comorbid anxiety, substance abuse and other mental diseases, resulting in great damage to their ability to live, study and work. The vast majority of BD patients need lifelong monitoring and treatment. Due to the multi-etiological complexity of the disease, there are no specific biomarkers to guide clinical diagnosis and the evaluation of drug treatment effectiveness. Researchers have found that BD may be associated with changes in neurotransmitters, immunology, neuroimaging, neuroendocrinology, neuroplasticity, molecular genetics, and adverse environmental stress (4–8), but they have not been conclusively consistent. Therefore, the study of objective markers of BD has always been a hot and difficult research topic.

Although the pathologic mechanisms of BD are poorly understood, the research on drugs to treat BD has made relatively considerable progress. At present, the commonly used emotional stabilizers for the treatment of BD are lithium, valproate, lamotrigine and some antipsychotics (such as quetiapine and olanzapine) which have the effect of stabilizing emotion. Patients with BD at the onset of depression may be treated with a combination of antidepressants when mood stabilizers or antipsychotics do not work well. Interestingly, the therapeutic effect of these drugs on BD is mostly the conclusion of clinical observation and research, but their mechanism of action has not been clarified. Myo-inositol depletion is a well-characterized effect of lithium treatment and has also been found to occur after valproate treatment (9, 10). Antipsychotics and antidepressants work by acting on specific receptors. These limited findings cannot elucidate the internal mechanism of BD remission. Therefore, it will be a significant research direction to explore the pathology of BD from the perspective of the common pathway of the efficacy of drug therapy.

Metabolomics is a research method for qualitative and quantitative analysis of small molecular metabolites in biological samples (11), which is widely used to study the pathological mechanism of complex diseases, such as cancer, diabetes, cardiovascular diseases, and psychiatric diseases, as well as to find new biomarkers for diagnosis and prognosis. Many studies on the metabolomics of BD have been reported, mainly including the study on the diagnostic biomarkers of BD and the changes of metabolites related to drug treatment (12–15). These studies have found some significant specific metabolites, which need to be verified by more similar studies. Studies on biomarkers related to the efficacy of drug therapy for BD are rarely reported, and most of them are *in vitro* studies, such as animal models or post-mortem brain.

Considering that the depression episode is more pervasive than manic states in the course of BD (16), and the pathological mechanism of these two mood polarities are different, this research focused on bipolar depression. As defined in DSM-5, depression is characterized by at least 2 weeks of depressive mood and/or anhedonia, along with at least four of other possible symptoms that include changes in energy, psychomotor activity, concentration, sleep, appetite/weight, thought content (guilt and worthlessness) and suicidal intent. Our research group has carried out a previous study on diagnostic biomarkers in the blood of bipolar depression and found some differential metabolites (17). We would like to further investigate how the specific differential metabolites of bipolar depression at baseline will change as their condition improves with medication.

Therefore, in this study, patients with bipolar depression, belonging to BD II type, were treated with medication for 8 weeks, and the changes of plasma metabolites before and after medication were analyzed using ^1^H-NMR metabolomics analysis method, so as to screen the metabolites that can be used for the evaluation of drug treatment effectiveness and analyze the internal mechanism of disease improvement by drug therapy.

## Materials and Methods

### Subjects Recruitment and Sample Collection

Ethics approval for this study is held by the medical ethics committee of Shanxi Bethune hospital (the Approval Notice Number: YXLL-2020-001). All subjects enrolled in the study gave their written informed consent. In order to reduce the interference of different types of BD on the results, the inclusion requirements of the patient group were first to conform to the BD II type, which is characterized by the intermittent occurrence of threshold depressive episodes and hypomanic episodes that are qualitatively like manic periods but are not of a sufficient duration or severity to cause significant functional impairment, hospitalization, or psychosis. Thirty-one unmedicated patients with bipolar depression were eventually recruited from the outpatient and inpatient of Shanxi Bethune Hospital and completed 8 weeks of drug therapy and follow-up, and 47 healthy subjects matching their demographic characteristics were also recruited from the hospital's physical examination center. All subjects were between 18 and 60 years old. The patients in the bipolar depression group were diagnosed by the Diagnostic and Statistical Manual of Mental Disorders V (DSM-V). Patients with current or previous presence of any physical or other mental disorders, as well as substance abuse were excluded. The subjects in HC group should also have no physical or mental disorders, and substance abuse.

General demographic data, including sex, age, marital status, and body mass index (BMI), were recorded for the patient group and the healthy control group. Subjects with BD were assessed using the 24-item Hamilton Depression Rating Scale (HAMD-24), 14-item Hamilton Anxiety Rating Scale (HAMA-14) and Clinical General Impression Rating Scale (CGI) at baseline and post-8 weeks medication. In this study, the formulation of the medication regimens were referred to the pharmacological treatment principles of acute bipolar depression in *The Chinese Guidelines for the Prevention and Treatment of Bipolar Disorder (Second Edition) and Canadian Network for Mood and Anxiety Treatments (CANMAT) and International Society for Bipolar Disorders (ISBD) 2018 guidelines for the management of patients with bipolar disorder* (18). Single drug therapy was the first choice for the treatment of acute bipolar depression (such as quetiapine, lithium, and Lamotrigine). Once the effect of monotherapy is not ideal, the combination of drugs should be considered, which means the combination of mood stabilizers (such as lithium, Lamotrigine and valproate) and/or atypical antipsychotics (such as quetiapine and olanzapine). Treatment with the combinations of an antidepressant [selective serotonin reuptake inhibitors (SSRIs) or bupropion] on the basis of lithium/divalproex or an atypical antipsychotic is considered second-line medication regimes. Antidepressants should be used with caution in patients with a history of antidepressant-induced mania or hypomania. Efficacy was assessed according to the reduction rate of HAMD (cure: a reduction of HAMD scores by Z75%; significant progress: a reduction of Z50%; progress: a reduction of Z25%; ineffective: a reduction of <Z25%).

### Plasma Collection

Blood samples of the recruited subjects were collected using vacuum blood collection vessels containing heparin sodium by professionals in the morning (between 07:30 and 10:00) after a 12-h fasting. The blood samples were mixed upside down to prevent coagulation, kept for 0.5 h at room temperature, and then centrifuged for 15 min at 1,600 × g. The obtained supernatant was sucked out into a sterile EP tube with a pipette and stored at −80°C until NMR analysis. The plasma of healthy controls was collected once, and the plasma samples of bipolar depression subjects were collected before and after 8 weeks of medication.

### Sample Preparation and NMR Procedure

The plasma samples should be pretreated before NMR analysis. The pre-processing flow is as follows: (1) The plasma samples were removed from the −80°C refrigerator and thawed in the ice water compound; (2) 450 ul plasma samples were extracted with a pipette and mixed with 350 ul phosphate buffer (0.2 M Na2HPO4/NaH2PO4, pH = 7.4) in D2O containing sodium 3-trimethylsilyl-(2, 2, 3, 3-d4)-1-propionate (TSP, 0.01%); (3) The mixture was centrifuged for 20 min at 15,493 × g at 4°C; (4) The supernatant (600 μl) was transferred into 5 mm NMR tubes for NMR analysis.

The Bruker 600 MHz AVANCE III NMR spectrometer (Bruker Biospin, Rheinstetten, Germany) was used for data acquisition. The ^1^H NMR spectra of the plasma samples were recorded in a one-dimensional (1D) Carr–Purcell–Merboom–Gill [CPMG, RD−90-(τcp-180-τcp)-acquisition] with water suppression. The key parameters for the NMR procedure were as follows: ^1^H frequency, 600.13 MHz; test temperature, 293 K; scanning time/scans, 5 min/64; spectral width, 12,019.2 Hz; spectral size, 65,536 points; pulse width (PW), 30 (12.7 μs); relaxation delay (RD), 1.0 s; The free induction decay (FID) was Fourier transformed with a line-broadening factor of 0.3 Hz. The NMR spectra of plasma samples were acquired according to previous relevant literature and NMR databases (19).

### Data Processing

Phase and baseline adjustment of the acquired ^1^H NMR spectra was performed manually using the MestReNova software (Mestrelab Research, Santiago de Compostella, Spain). The chemical shift of creatinine (δ 3.04 ppm) was used as a calibration standard. The region of δ 4.7–5.2 ppm was excluded due to residual water. Each spectrum was then segmented at δ 0.01 intervals across the region of δ 0.5–5.5 ppm. These data of integral value were finally normalized by dividing an individual peak area by the total peak area in ^1^H NMR profile of each subject for further multivariate analysis.

### Statistical Analyses

The processed spectra data were imported into SIMCA-P 14.1 software (Umetric, Sweden) for Orthogonal Partial Least Square Discriminant Analysis (OPLS-DA). The score plot of the OPLS-DA model could be used to visualize the differences between group clusters. It is necessary to perform 200 permutation tests and evaluate the validity of the established OPLS-DA model according to the values of *R*^2^ and *Q*^2^. The corresponding loading plot of the OPLS-DA model was further established to discover the differential metabolites contributing to samples separation. The differential metabolites were finally identified according to the standard of variable importance in the projection (VIP > 1) in the loading plot and *p* < 0.05 of independent samples *t*-test between BD at baseline and healthy controls (HCs) or *p* < 0.05 of paired samples *t*-test between BD patients at baseline and BD patients after 8 weeks medication using SPSS 25 software.

To determine the pathways involved in relative metabolites, they were further introduced into MetaboAnalyst 4.0 to perform Pathway analysis by selecting the human Pathway library. Pathways were screened according to the *p-*values of pathway enrichment and impact values of pathway topology analysis.

## Results

### General Information of Subjects

Thirty-one patients with bipolar depression were enrolled and completed 8 weeks of medication. Table 1 shows the clinical features of the subjects. Age, gender, BMI and marital status between BD patients at baseline and HCs were compared using Chi square test or independent samples *t*-test, and the results indicated no significant differences (*p* > 0.05). The clinical features between BD patients at baseline and BD patients after 8 weeks medication was compared using paired samples *t*-test, and results are shown in Table 1. Depression, anxiety and clinical status of patients with bipolar depression at baseline were shown by HAMD scores, HAMA scores and CGI scores. These patients did not meet the diagnosis of anxiety disorder. After 8 weeks medication, HAMD scores, HAMA scores and CGI scores of patients in BD group were significantly reduced, and the reduction rate of the three scales was above 50% which indicated significant curative effect and significant improvement of mood symptoms of BD subjects. All factor scores of HAMD and HAMA were also significantly reduced after 8 weeks of drug therapy.

**Table 1:** General characteristics of the subjects, including BD patients at baseline and after 8 weeks medication, and healthy controls.

**Clinical features**	**BD** **(***n*** = 31)^b^**		**Healthy controls** **(***n*** = 47)^b^**		
	**Baseline**	**After 8 weeks medication**		***p-*** **value^c^**	***p-*** value^*^^c^
Age (years)	30.26 ± 12.695	—^d^	30.66 ± 8.231	—	0.867
Gender(male/female)	13/18	—	23/24	—	0.644
BMI (kg/m^2^)^a^	23.42 ± 3.766	—	22.31 ± 3.041	—	0.177
Spouse (with/without)	19/12	—	23/24	—	0.355
HAMD-24 total score^a^	21.42 ± 7.86	9.13 ± 7.293	—	0.000	—
**HAMD factor score**				
Anxiety/somatization	5.45 ± 2.474	2.68 ± 1.759	—	0.000	—
Weigh reduction	0.16 ± 0.374	0.06 ± 0.250	—	0.235	—
Cognitive impairment	3.94 ± 2.351	2.03 ± 2.008	—	0.001	—
Diurnal variation	0.71 ± 1.039	0.29 ± 0.643	—	0.419	—
Slowness	4.61 ± 2.108	2.29 ± 1.736	—	0.000	—
Sleep disorder	2.23 ± 1.687	1.03 ± 0.875	—	0.001	—
Despair sense	4.16 ± 1.791	1.32 ± 1.720	—	0.000	—
HAMA-14 total score^a^	16.58 ± 6.076	8.00 ± 5.373	—	0.000	—
**HAMA factor score**				
Anxiety of somatization	10.45 ± 4.146	5.55 ± 3.641	—	0.000	—
Mental anxiety	5.48 ± 2.839	2.65 ± 2.229	—	0.000	—
CGI score^a^	4.55 ± 0.888	2.90 ± 0.790	—	0.000	—
**Medicine**				
Mood stabilizers	28 (90.32%)	—	—	—	—
Lithium	17 (54.84%)	—	—	—	—
Lamotrigine	9 (29.03%)	—	—	—	—
Sodium valproate	2 (6.45%)	—	—	—	—
Antipsychotics	22 (70.97%)	—	—	—	—
Antidepressants	7 (22.58%)	—	—	—	—

a *HAMD-24, 24-item Hamilton Depression Rating Scale; HAMA-14, 14-item Hamilton Anxiety Rating Scale; CGI, Clinical global impression scale; BMI, Body mass index*.

b *Values expressed as the mean ± SD (range)*.

c p-value represent BD patients at baseline vs. BD patients after 8 weeks medication, and p-value^*^ represent BD at baseline vs. Healthy controls.

d *“–” Means that the data are not shown*.

### Identification of Metabolites in the ^1^H-NMR Profiles

**Figure 1:**
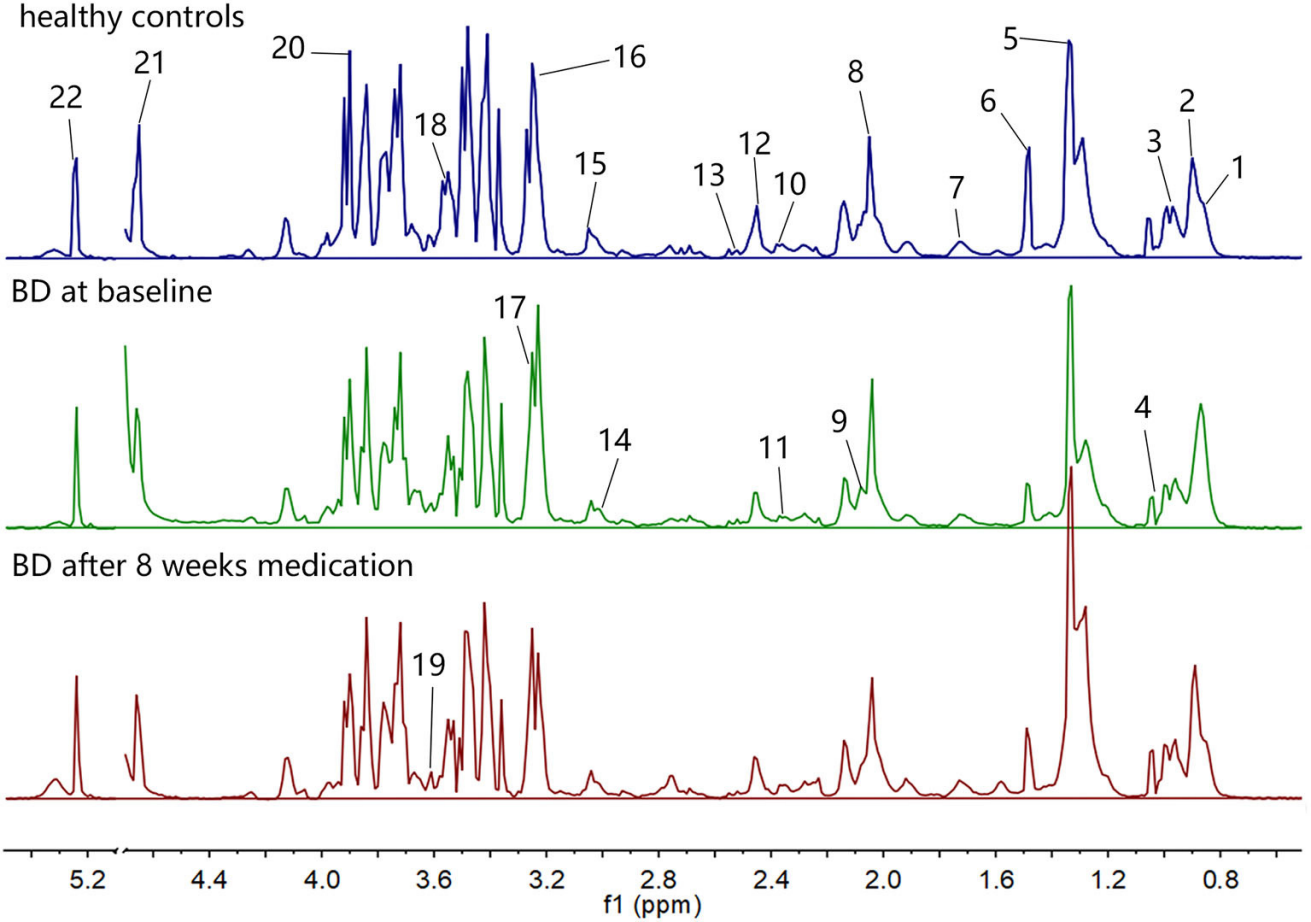
The typical ^1^H NMR spectra of the three groups of plasma samples.

The ^1^H NMR spectra of the three groups of plasma samples were detected one by one. Figure 1 shows the typical ^1^H NMR spectrum profiles of BD patients at baseline and after 8 weeks medication, and HCs. In the typical ^1^H NMR spectra of the three groups, a total of 22 metabolites were identified by looking up the Human Metabolome Database (HMDB) (http://www.hmdb.ca/) and related articles over the years (20, 21). The chemical shift values of these 22 metabolites in the typical ^1^H NMR spectra are listed in Table 2.

**Table 2:** Peak attribution in the typical ^1^H-NMR spectra of plasma samples.

**No**.	**Metabolites**	**Chemical shift (δ)**
1	Lipid	0.86 (t), 1.28 (m), 1.58 (m), 2.01 (m),
2	Isoleucine	0.93 (t, *J* = 7.41 Hz), 1.01 (d, *J* = 7.01 Hz)
3	Leucine	0.96 (t, *J* = 7.08 Hz)
4	Valine	0.99 (d, *J* = 8.41 Hz), 1.04 (d, *J* = 8.46 Hz)
5	Lactate	1.33 (d, *J* = 8.40 Hz), 4.11(q, *J* = 8.32 Hz)
6	Alanine	1.48 (d, *J* = 8.57 Hz), 3.77 (m)
7	Lysine	1.72 (m),1.90 (m)
8	N-acetyl glycoprotein	2.04 (s)
9	Glutamate	2.08 (m), 2.35 (m), 3.75 (m)
10	β-hydroxybutyric	2.34 (dd)
11	Pyroracemic	2.37 (s)
12	Glutamine	2.45 (m)
13	Citrate	2.53(d, *J* = 18.19 Hz), 2.65(d, *J* = 18.19 Hz)
14	Creatine	3.02 (s)
15	Creatinine	3.04 (s), 3.91 (s)
16	Choline	3.21 (s), 3.51 (t, *J* = 2.20 Hz)
17	Trimethylamine oxide	3.26 (s)
18	Glycine	3.56 (s)
19	Threonine	3.60 (d, *J* = 4.8 Hz), 4.24 (m)
20	Aspartic	3.90 (dd)
21	β- glucose	4.65 (d, *J* = 7.80 Hz)
22	α- glucose	5.24 (d, *J* = 4.20 Hz)

### Multivariate Statistical Analysis

**Figure 2:**
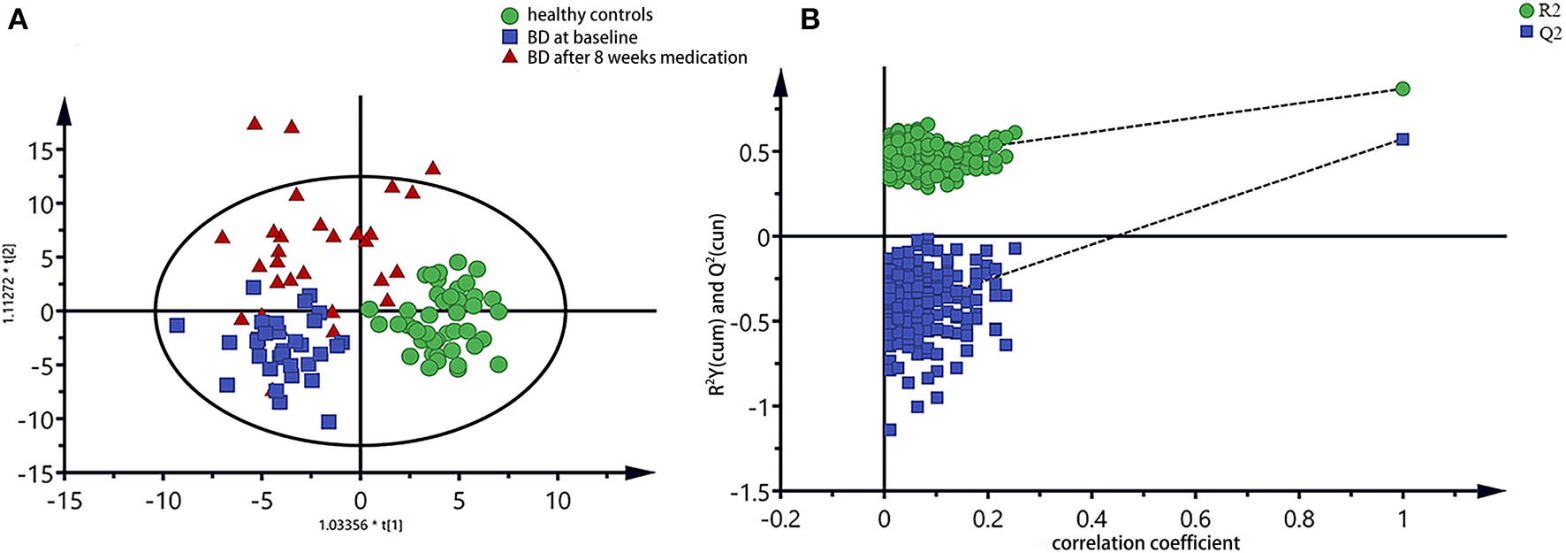
PLS-DA model of all three groups of plasma spectra, **(A)** PLS-DA score plots of ^1^H NMR spectra, in which each point represented a plasma metabolome of one subject, and the distance between data points reflected the scale of their metabolic differences; **(B)** 200-iteration permutation test map of the PLS-DA model.

The integral data of all the plasma spectra were taken into the SIMCA-P 14.1 software (Umetric, Sweden) for multivariate statistical analysis. We established the PLS-DA model to observe vividly metabolites differences of the three groups. The score plot of the PLS-DA model showed clear differentiations among the group of bipolar depression at baseline, the group of bipolar depression after 8 weeks medication and HC group, and that the bipolar depression after 8 weeks medication group was closer to the HC group than bipolar depression at baseline group, suggesting a disturbance of the plasma metabolites in bipolar depression patients and a trend to reversion to the healthy control state after 8 weeks medication (Figure 2A). Permutation testing showed that the cumulative *R*^2^- and *Q*^2^-values were all less than the original value, indicating that the PLS-DA model was not over-fitted (Figure 2B).

**Figure 3:**
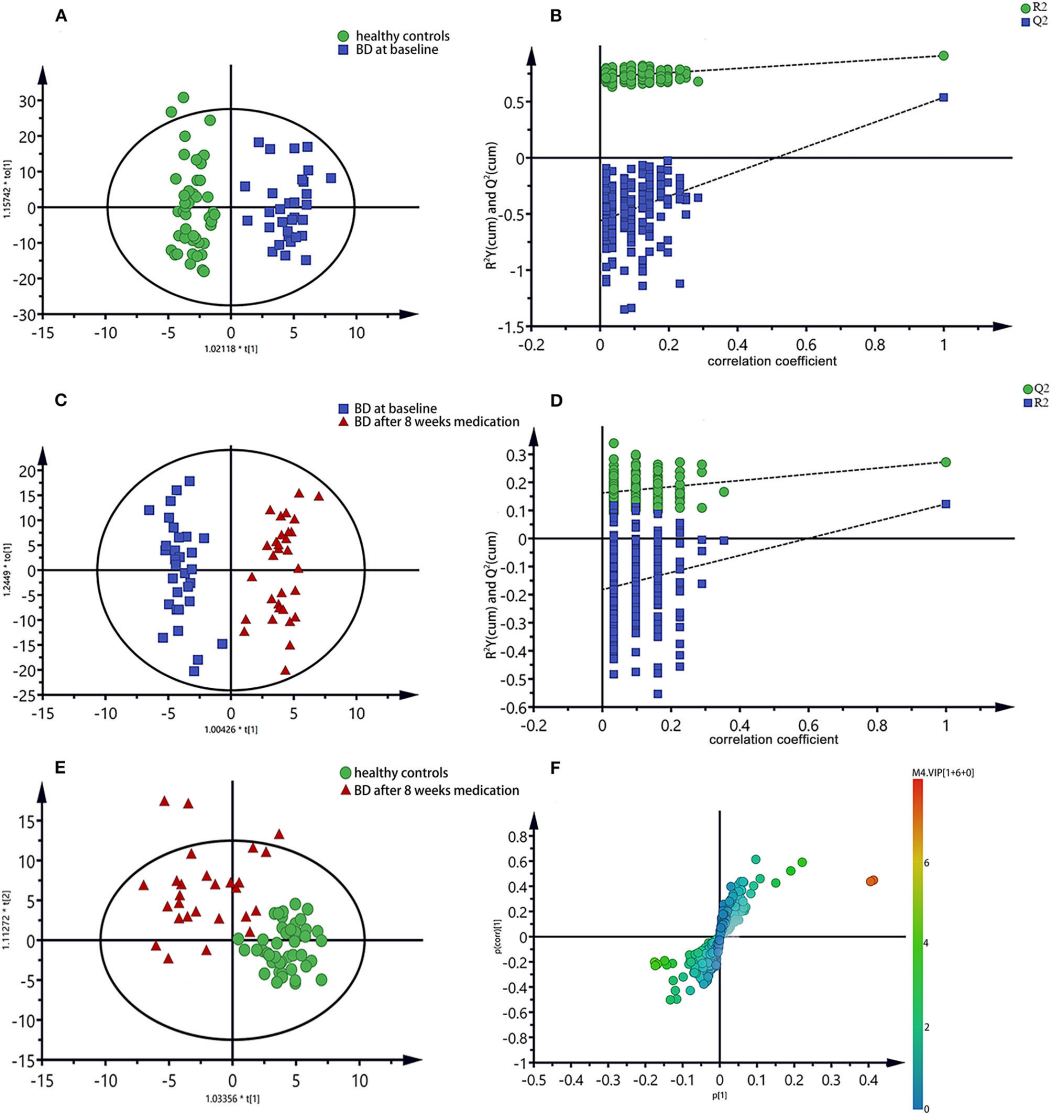
OPLS-DA scores plots of plasma samples **(A,C,E)**, the permutation test maps **(B,D)** and the corresponding S-plots **(F)**, **(A,B)**: healthy controls vs. bipolar depression patients at baseline; **(C,D,F)**: bipolar depression patients at baseline vs. bipolar depression patients after 8 weeks medication; **(E)**: healthy controls vs. bipolar depression patients after 8 weeks medication.

**Figure 4:**
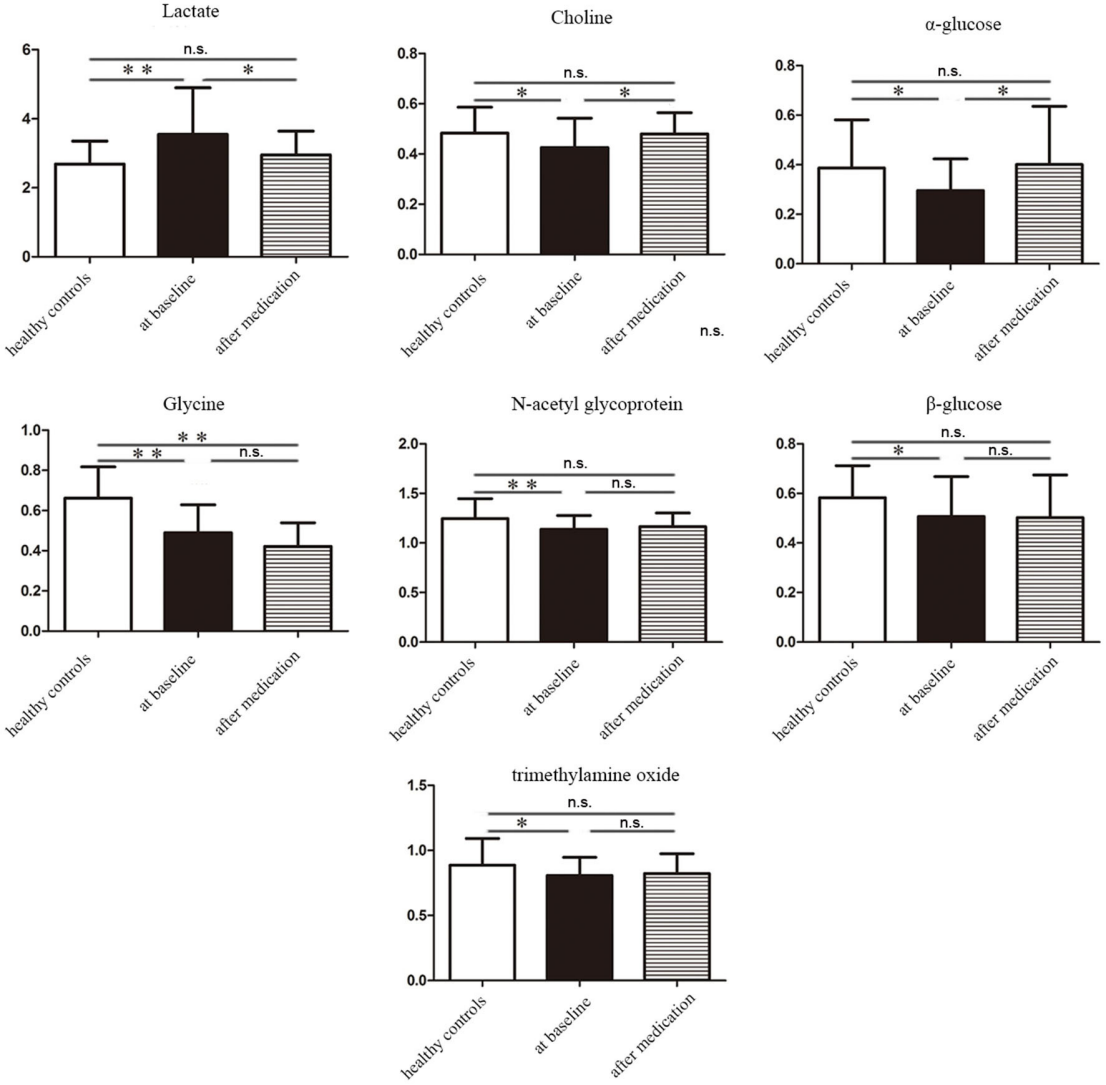
The concentration variation diagram of the 7 different metabolites among healthy controls, bipolar depression patients at baseline and bipolar depression patients after 8 weeks medication. n.s., non-significant ^*^P < 0.05, ^**^P < 0.01.

To further identify the metabolites changes associated with bipolar depression at baseline and after 8 weeks medication, OPLS-DA was applied in this study. The group of bipolar depression at baseline was significantly separated from HC group in the OPLS-DA scores plot (Figure 3A), which were verified to be valid by the permutation testing (Figure 3B). Consistent with our previous findings, bipolar depression patients at baseline were characterized by significantly higher levels of lactate, and significantly lower levels of Choline, α-glucose, β-glucose, glycine, N-acetyl glycoprotein, trimethylamine oxide compared to HCs (17). Similarly, the OPLS-DA scores plot between the group of bipolar depression at baseline and the group of bipolar depression after 8 weeks medication was established (Figure 3C), which showed a clear separation between the two groups and were verified to be valid by the permutation testing (Figure 3D). The score plot of OPLS-DA model showed no significant difference between healthy controls and bipolar depression after 8 weeks medication, with some overlap. This indicates that the metabolite of BD after 8 weeks medication differs little from that of healthy controls (Figure 3E). The corresponding S-plot (Figure 3F) indicated that the lactate, choline, α-glucose, β-glucose, N-acetyl glycoprotein and trimethylamine oxide had the tendency to return to the normal levels after 8 weeks medication (VIP > 1 and *p* < 0.05), and especially the Lactate, choline and α-glucose could be basically restored to normal levels. The changes of these 7 metabolites between the three groups were clearly shown in Figure 4. These results mean that effective medication had significant effects on plasma metabolites of bipolar depression.

### Pathways Analysis of Metabolites Related to Medication

**Figure 5:**
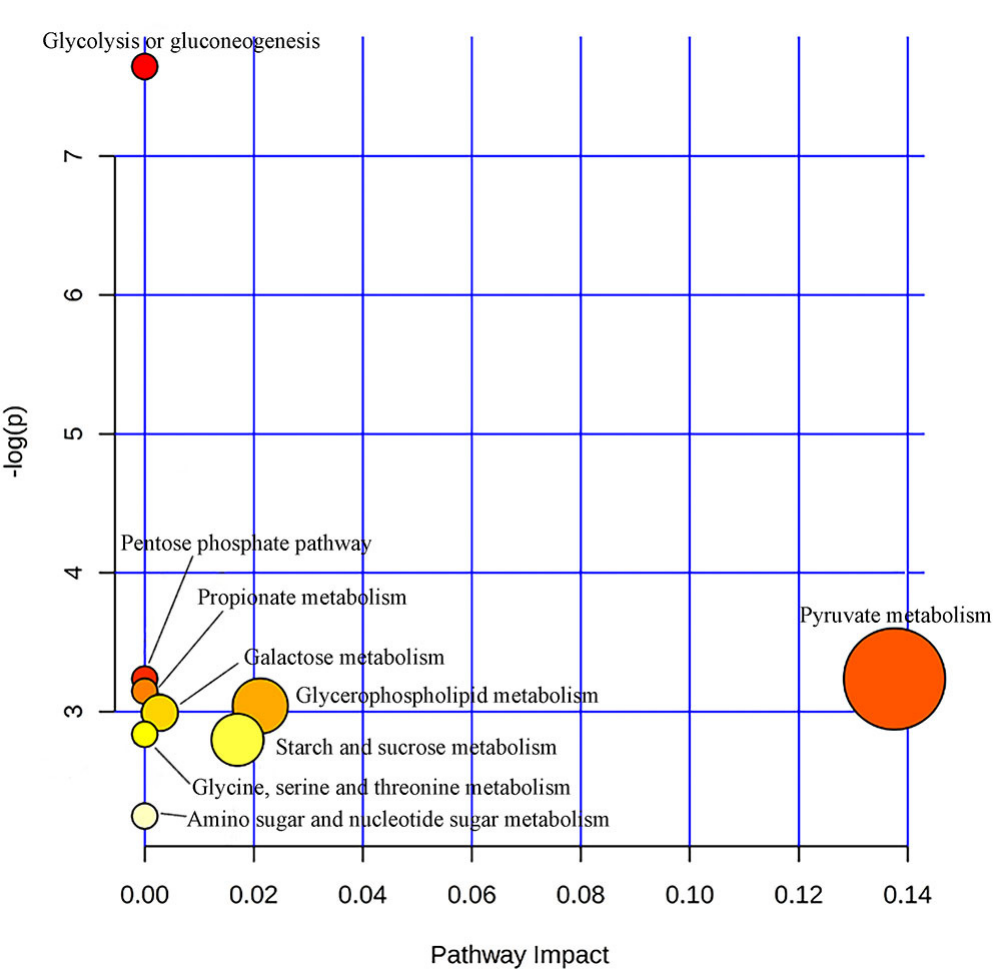
Metabolic pathways analysis map of the metabolites related to the efficacy of medication for bipolar depression. Color intensity (white to red) reflects increasing statistical significance, while circle diameter covaries with pathway impact.

In order to further explore the mechanism of the efficacy of medication for bipolar depression, the metabolic pathways involved in these different metabolites related to the efficacy of medication were analyzed. The main metabolic pathways involved were: (1) pyruvate metabolism, (2) glycolysis or gluconeogenesis, (3) glycerophospholipid metabolism, (4) starch and sucrose metabolism, (5) pentose phosphate pathway, (6) propionate metabolism, (7) galactose metabolism, (8) glycine, serine and threonine metabolism, and (9) amino sugar and nucleotide sugar metabolism (Figure 5).

## Discussion

This study is a continuation of our group's research on blood diagnostic biomarkers for BD. We excluded patients who did not belong to bipolar II type and those who were not willing to participate in the follow-up. A total of 31 unmedicated patients in the acute stage of bipolar depression were recruited for 8 weeks of medication and follow-up. Encouragingly, the results of differential metabolites associated with bipolar depression were consistent with the previous results, which to some extent verified the reliability of our previous study results. As for the detection technique of metabolites profiles, we still chose a relatively mature ^1^H-NMR metabolomics technique. This single technique was able to detect a limited number of metabolites, and we tried to look for differential metabolites related to diagnosis and efficacy of medication of bipolar depression in these limited metabolites range.

The efficacy of medication was assessed according to the reduction rate of HAMD. In the absence of objective biological indicators for the assessment of mental illness, the HAMD scale, characterized by its relative objectivity and convenience, is the main clinical method for the assessment of depression severity and efficacy of medication. In this study, HAMD scores were used to help determine the acute phase and the effectiveness of medication in patients with bipolar depression. And the metabolite profiles were compared between the acute and significant improvement stages of bipolar depression based on HAMD score to look for changes of blood metabolites regardless of specific drug regimen.

The results of this study were that patients with bipolar depression showed relatively consistent changes in blood metabolites after 8 weeks medication. Therefore, we conclude that the intrinsic biological changes in significant improvement stage of bipolar depression are likely to be consistent despite the different medication regimens. Previous studies have reported that both lithium and valproate can reduce inositol levels in BD patients for therapeutic effects (22). These metabolites that change with significant improvement were related to energy metabolism (lactic acid, α-glucose, β-glucose), choline, amino acid metabolism (glycine, N-acetyl glycoprotein), and intestinal microbial metabolism (trimethylamine oxide), the levels of which had the tendency to return to the normal after 8 weeks medication. Only Glycine was reduced in both the acute and significant improvement stages of BD, suggesting that glycine may be a characteristic marker of bipolar depression and does not change with the improvement of bipolar depression. These altered metabolites involved multiple metabolic pathways, and pyruvate metabolism was more closely related to therapeutic effect evaluation of BD (pathway impact > 0.1,*p* < 0.05).

Energy metabolism is one of the most important metabolic ways in the body. In this study, we observed significant decreases in α-glucose and β-glucose levels and significant increases in lactic acid levels in patients with bipolar depression at baseline. After 8 weeks medication, the levels of α-glucose and lactic acid were basically normal, and the level of β-glucose also has the tendency to return to normal. Glucose is the main source of energy in the body, and its aerobic oxidation is the main way of energy supply. In the absence of oxygen supply, sugar goes through anaerobic oxidation to produce lactic acid, leading to a decline in energy supply (23). Therefore, the results of this study suggested that the anaerobic oxidation pathway was enhanced and the aerobic oxidation pathway was inhibited in patients with bipolar depression. This energy metabolism disorder in bipolar depression may explain the symptoms of fatigue, poor energy and slow thinking. Drug therapy can regulate energy metabolism and restore it to normal. Energy homeostasis imbalances of BD have been previously reported (24, 25).

Choline is an important substance in the body, which is the main synthetic raw material of glycerol phospholipids and can also be used in the synthesis of acetylcholine, which is the important neurotransmitter (26). In this study, patients with bipolar depression had reduced choline levels and returned to normal after 8 weeks of medication. Reduced choline levels in patients with bipolar depression can lead to reduced production of glycerophospholipids and acetylcholine, both of which are important functional substances. Effective drug therapy could improve biological function by increasing blood choline levels. Previous studies have found abnormal levels of choline in BD (27). Therefore, serum choline level is expected to be a biomarker for the diagnosis and the evaluation of drug treatment effectiveness of bipolar depression.

This study found that patients with bipolar depression had lower glycine levels than HCs, and glycine levels continued to decrease after effective medication. The results indicated that glycine may be a characteristic marker of bipolar depression and does not change with the improvement of bipolar depression. Glycine can affect the excitability of the nervous system (28, 29). It has been reported that glycine, serine and threonine have obvious changes in serum of patients with refractory depression (30). Changes in glycine levels were also found in the cerebrospinal fluid of patients with BD (31). Therefore, it can be speculated that glycine is abnormal in some mental disorders, not just bipolar disorder and may be involved in the pathological mechanism of psychiatric diseases. The study also found that baseline levels of N-acetylamino glycoprotein were lower in patients with bipolar depression than in HCs, and returned to normal after medication. Due to the single technology in this study, n-acetylamino glycoprotein classification could not be carried out, so future research could improve on this.

We also found elevated trimethylamine oxide levels in patients with bipolar depression, which returned to normal after 8 weeks of medication. Trimethylamine oxide is a metabolite related to intestinal flora, and its production cannot be separated from the involvement of intestinal flora. Intestinal flora is closely related to human health (32). Researchers have found that gut flora can affect human brain function through the microbiota-gut-brain axis (33). Several studies in recent years have reported abnormal intestinal flora in BD (34–36). Combined with these findings, we believe that intestinal flora abnormalities and their metabolic disorders may be one of the pathological mechanisms of BD.

Although some significant results were found in this project, we are aware of some limitations of this research and inspired with some prospects for future research. Firstly, we found differential metabolites related to bipolar depression and curative effect from relatively few research samples, and no quantitative analysis of these differential metabolites was conducted. In the future, a normal numerical range and abnormal range could be established in combination with a large number of similar studies, for further effective clinical application. Secondly, the medication regimen of the BD patients was formulated according to the clinical guidelines, and most of them were combined medication, so it was difficult to determine the effect of a single drug on plasma metabolites of the BD patients. Thirdly, There was no correlation analysis between metabolites and symptom severity. Therefore, the correlation between differential metabolites and BD symptoms did not be intuitively demonstrated.Fourthly, the number of differential metabolites might be not enough to obtain the robust results of pathway analysis and enrichment analysis. Fifthly, all subjects were from the same location and might share the same dietary habits, which may restrict the generalization of the findings. Last, in the future, dynamic monitoring of metabolite changes in each stage of BD can be carried out, which is conducive to in-depth analysis of its pathological evolution.

In general, we found that certain meaningful blood metabolites were associated with the diagnosis and evaluation of drug treatment effectiveness of bipolar depression. Lactate was elevated during the acute phase of bipolar depression and basically returned to normal levels after 8 weeks of effective medication. Levels of Choline, α-glucose, β-glucose, N-acetyl glycoprotein and trimethylamine oxide all decreased in the acute phase of bipolar depression and returned to normal levels to varying degrees in significant improvement stage. Glycine continued to decrease at baseline and after 8 weeks medication of bipolar depression. Therefore, it can be speculated that glycine may be a characteristic marker of bipolar depression and does not change with the improvement of bipolar depression, while lactate, choline, α-glucose, β-glucose, N-acetyl glycoprotein and trimethylamine oxide may be biomarkers associated with the pathological development of bipolar depression and can reflect the severity of the illness. These identified metabolites were associated with multiple metabolic pathways, especially pyruvate metabolism. Moreover, these metabolites are involved in energy metabolism, amino acid metabolism and intestinal microbial metabolism, which were consistent with some previously reported studies. Metabolomics has a great development prospect in the study of biological indicators related to the efficacy of BD. It is hoped that more studies in this field with a larger sample size will be carried out in the future, so as to further explore the internal mechanism of the course evolution of BD.

## Data Availability Statement

The original contributions presented in the study are included in the article/supplementary material, further inquiries can be directed to the corresponding author/s.

## Ethics Statement

The studies involving human participants were reviewed and approved by the Medical Ethics Committee of Shanxi Bethune Hospital. The patients/participants provided their written informed consent to participate in this study.

## Author Contributions

YR conceived and designed the experiments. JJ, X-HC, SB, FY, L-NM, and X-XC collected subjects. PW performed the experiments and analyzed the data. X-JG, PW, and YR wrote and revised the manuscript. All authors contributed to the article and approved the submitted version.

## Funding

This work was supported by the National Natural Science Foundation of China (8210053813), Applied Basic Research Projects of Shanxi Province, China (201901D111418), Research Project Supported by Shanxi Scholarship Council of China (2021-167).

## Conflict of Interest

The authors declare that the research was conducted in the absence of any commercial or financial relationships that could be construed as a potential conflict of interest.

## Publisher's Note

All claims expressed in this article are solely those of the authors and do not necessarily represent those of their affiliated organizations, or those of the publisher, the editors and the reviewers. Any product that may be evaluated in this article, or claim that may be made by its manufacturer, is not guaranteed or endorsed by the publisher.

## Abbreviations

BD: Bipolar disorder
HC: healthy control
NMR: Nuclear magnetic resonance
PLS-DA: Partial Least Square Discriminant Analysis
OPLS-DA: Orthogonal Partial Least Square Discriminant Analysis
DSM-V: The Diagnostic and Statistical Manual of Mental Disorders V
VIP: Variable importance in the projection
HAMD-24: 24-item Hamilton Depression Rating Scal
HAMA-14: 14-item Hamilton Anxiety Rating Scale
CGI: Clinical General Impression Rating Scale
BMI: Body mass index.
